# Large Language Models for Real-World Nutrition Assessment: Structured Prompts, Multi-Model Validation and Expert Oversight

**DOI:** 10.3390/nu18010023

**Published:** 2025-12-20

**Authors:** Aia Ase, Jacek Borowicz, Kamil Rakocy, Barbara Piekarska

**Affiliations:** 1 Department of Internal Medicine, Hypertension and Vascular Diseases, Medical University of Warsaw, 02-097 Warsaw, Poland; 2 Department of the Prevention of Environmental Hazard, Allergology and Immunology, Faculty of Health Sciences, Medical University of Warsaw, 02-091 Warsaw, Poland; jacek.borowicz@wum.edu.pl (J.B.); barbara.piekarska@wum.edu.pl (B.P.); 3 KR Consulting, 02-601 Warsaw, Poland

**Keywords:** large language models, dietary management, artificial intelligence in nutrition, artificial intelligence in healthcare, digital tools, nutrition estimation

## Abstract

**Background:** Traditional dietary assessment methods face limitations including reporting bias and scalability challenges. Large language models (LLMs) offer potential for automated food classification, yet their validation in morphologically complex, non-English languages like Polish remains limited. **Methods:** We analyzed 1992 food items from a Polish long-term care facility (LTCF) cohort using three advanced LLMs (Claude Opus 4.5, Gemini 3 pro, and GPT-5.1-chat-latest) with two prompting strategies: a structured double-step prompt integrating NOVA and World Health Organization (WHO) criteria, and a simplified single-step prompt. Classifications were compared against consensus judgments from two human experts. **Results:** All LLMs showed high agreement with human experts (90.3–94.2%), but there were statistically significant differences in all pairwise comparisons (χ^2^ = 1174.5–1897.1; *p* < 0.001). The structured prompt produced very high Recall for UNHEALTHY items at the cost of lower Specificity, whereas the simplified prompt achieved higher overall Accuracy and a more balanced Recall–Specificity profile, indicating a trade-off between strict guideline adherence and alignment with general human judgment. **Conclusions:** Advanced LLMs demonstrate near-expert accuracy in Polish-language dietary classification, enhancing workflow efficiency by shifting effort toward validation. Expert oversight remains essential, and multi-model consensus alongside language-specific validation can improve AI reliability in nutrition assessment.

## 1. Introduction

### 1.1. Advances in Artificial Intelligence for Nutrition Science

Accurate dietary assessment is essential for nutritional epidemiology, clinical nutrition, and public health initiatives to reduce the worldwide impact of diet-related chronic diseases [1]. Conventional assessment tools, such as food frequency questionnaires, 24-h recalls, and weighed food records, have limitations like participant burden and reporting biases, which diminish their overall usability and accuracy [2]. Recent advances in artificial intelligence (AI), particularly natural language processing and large language models (LLMs), offer promising avenues for automating nutritional assessment [3]. These AI-driven technologies provide scalability and improved precision by analyzing complex free-text dietary data and conducting high-throughput nutrient and food classification. They also support personalized nutrition recommendations, representing a paradigm shift in the field of nutritional sciences [4,5].

### 1.2. The Role of Large Language Models in Dietary Classification

LLMs like OpenAI’s GPT series, Anthropic’s Claude, and Google’s Gemini are at the forefront of AI architecture, enabling advanced reasoning, contextual understanding, and structured data processing. Their emerging usefulness spans clinical and consumer nutrition, including food item classification, nutrient measurement, and dietary quality assessment [6,7]. However, most validation studies focus on English-language datasets, raising questions about the performance and cultural adaptation of these LLMs when applied to morphologically rich, complex languages like Polish. Addressing this gap is essential for developing culturally and linguistically aware AI nutrition applications [8,9].

### 1.3. Artificial Intelligence, Large Language Models, and Real-World Dietary Data

AI, especially LLMs, aims to transform dietary assessment through scalable, automated analysis of complex dietary data [3,10,11]. However, linguistic complexity, cultural context, and the diversity of food environments, as seen in the diets and ‘off-menu’ items in Polish long-term care facilities (LTCFs), present unique challenges. These issues require careful evaluation of LLM suitability and thorough validation to ensure reliability and relevance [10].

### 1.4. The Impact of Ultra-Processed Foods on Dietary Quality and Health

Recent meta-analyses and systematic reviews consistently show that high consumption of ultra-processed foods (UPFs) is linked to increased risks of all-cause mortality, cardiovascular disease, diabetes, and mental health disorders [12,13]. These findings have energized worldwide debates on food processing, emphasizing UPFs as a key factor in the increasing burden of non-communicable diseases (NCDs) across high- and middle-income countries [14]. The rise in UPFs, known for their high energy density, poor nutrient quality, and aggressive marketing, has led to the widespread replacement of traditional minimally processed diets. This shift raises serious concerns for public health efforts [15].

### 1.5. Integrating Frameworks for Processing and Nutrient-Based Food Classification

Assessment of dietary quality typically relies on complementary frameworks: the NOVA system, which focuses on processing levels [16], and the World Health Organization (WHO) nutrient thresholds, which set quantitative limits on sugars, saturated fats, and sodium [17,18,19]. The interaction of these paradigms shapes global dietary guidelines but introduces methodological challenges when applied in AI systems. Evaluating AI performance across these frameworks improves our understanding of their combined strengths and limitations in large-scale dietary assessment [4,20].

### 1.6. Limitations and Critiques of the NOVA Classification

Despite its widespread use, the NOVA classification system has received significant critique within food science and nutrition research. Critics claim that the system’s reliance on processing levels and ingredient-based definitions creates broad, varied categories that may misrepresent the nutritional quality of individual foods [21,22]. For instance, some minimally processed foods prepared in industrial settings, or homemade foods with similar processing features, are misclassified as “ultra-processed,” possibly misleading consumers and policymakers. Additionally, binary classification oversimplifies complex food structures and nutrient profiles, underscoring the need for refined, hybrid systems that integrate processing and nutrient density for more precise evaluation [22,23].

### 1.7. Challenges and Opportunities in Multilingual AI Dietary Assessment

Real-world dietary datasets often include foods eaten outside structured institutional menus, such as off-menu items purchased or brought by individuals, which increases classification challenges [24,25]. The linguistic richness of Polish, a language recently recognized as highly suitable for LLM instruction-following [26], offers a uniquely rigorous testbed for evaluating AI models. This approach addresses geographic and cultural specifics that are rarely considered in current research [27].

### 1.8. Study Purpose and Significance

To address these gaps, the current study rigorously evaluates multiple advanced LLMs using a longitudinal Polish-language dataset of food items for LTCF residents. Employing an integrated framework that combines NOVA- and WHO-based classifications as a structured double-step prompt, along with a simplified single-step prompt, the study aims to measure inter-model agreement, examine the practical implications of classification criteria, and assess whether expert dietitian adjudication is necessary to ensure trustworthy AI integration in clinical and public health nutrition. By involving two independent human experts, this research establishes a robust gold-standard reference, enabling direct comparison of LLM performance with validated human judgment and strengthening the evidence for AI reliability in real-world nutritional assessment.

## 2. Materials and Methods

### 2.1. Study Design and Data Collection

This study used data from a comprehensive longitudinal project conducted between 2017 and 2021, funded by the Ministry of Health of Poland under the National Health Program 2016–2020 [28]. Ethical approval was obtained from the Institutional Ethical Review Board at the Medical University of Warsaw (Approval No. AK-BE/212/2017). The cohort included 1000 residents in LTCFs, from whom a comprehensive dataset was collected, including anthropometric parameters, bioimpedance measurements, physical activity records, clinical diagnoses, and detailed dietary intake information.

This investigation specifically analyzed food items stored in residents’ cabinets to assess dietary quality and compare how well different LLMs classify them. These items include foods purchased outside the officially prescribed diet, either by residents themselves or brought in by family members, and thus do not form part of the formal dietary plan provided by the care facility.

To process the dataset, we used three state-of-the-art LLMs, selected based on their release dates, computational costs, and efficiency with structured data:

- Claude Opus 4.5 (Anthropic)—Released on 24 November 2025;
- Gemini 3 pro (Google)—Released on 18 November 2025;
- GPT-5.1-chat-latest (OpenAI)—Released on 12 November 2025;

AI models were used in this study on 30 November 2025.

In total, 1992 food items were analyzed.

### 2.2. Double Step Prompt: Classification Frameworks Based on NOVA and WHO Guidelines

Food items were classified as either “healthy” or “unhealthy” through a standardized two-step method that integrates both NOVA classification and WHO guidelines:

1. Processing-Based Classification (NOVA System):

The NOVA classification system was used to identify UPFs, which are energy-dense and nutrient-poor. Items classified as UPFs were immediately labeled as unhealthy based on processing level.

2. Nutritional Threshold Evaluation (WHO Guidelines):

Items classified as healthy in Step 1 were further evaluated using WHO dietary thresholds. Products exceeding recommended limits for free sugars (>10% of total energy), saturated fats (>10%), or sodium (>2 g/day) were reclassified as unhealthy. To ensure accuracy and consistency, a structured AI-driven prompting framework was adopted. The LLMs were instructed to systematically assess food items, generating structured outputs including product name, weight (g), total caloric content, and final classification (healthy/unhealthy).

### 2.3. Prompting Framework and Data Processing

To evaluate LLMs’ agreement and classification consistency, we systematically examined discrepancies and documented:

- Food item classification (Healthy/Unhealthy)
- Justification for classification (based on NOVA and WHO criteria)

The final prompt was as follows:

“Prepare a dictionary with healthy and unhealthy food for each file and check it with this prompt: Step 1 Act as a helpful dietary assistant. Classify the following food products as ‘healthy’ or ‘unhealthy’ based on established nutritional criteria. For this classification, consider foods unhealthy if they are identified as ultra-processed according to the NOVA classification system. For each product, provide details in the following format: {product, weight (g), calories, classification}. Finally, sum the total calories for each category. Products: [list of products] Step 2—for all in HEALTHY: Act as a helpful dietary assistant. Classify the following food products as ‘healthy’ or ‘unhealthy’ based on established nutritional criteria. For this classification, consider foods unhealthy if they exceed WHO thresholds for free sugars (>10% of total energy), saturated fats (>10%), or sodium (>2 g/day). For each product, provide details in the following format: {product, weight (g), calories, classification}. Finally, sum the total calories for each category. Products: [list of products].”

### 2.4. Simplified Prompt

Building on the initial evaluation, which used an advanced two-step prompt combining the NOVA and WHO frameworks, an additional experiment was conducted to determine whether simpler instructions would yield similar or different results. To do this, we ran a classification round using the same dataset and the same LLMs, but with a simplified prompt. The simplified task asked the LLMs to evaluate each product and justify their choices briefly in a two-column format, as follows:

“Evaluate each product as healthy or unhealthy and explain why—in two columns: ‘evaluation’ and ‘description.’”

### 2.5. Evaluation by Human Experts

Additionally, a human expert independently classified the same database, assigning each item to either a healthy or unhealthy category and providing a brief justification for each decision. A second human expert then reviewed and validated these judgments to correct any possible errors, creating an optimized, high-accuracy reference standard for comparison with LLM performance. Human experts knew the database source but were instructed to evaluate items holistically and generally, without tailoring classifications to specific dietary restrictions.

### 2.6. Model Setup and Language Protocol

To ensure database consistency, all LLMs were accessed via API calls. Since Gemini’s official documentation strongly recommends keeping the default temperature parameter and warns that lowering it below 1.0 may lead to unexpected behaviors, such as response loops or reduced performance on complex reasoning tasks [29], all tested LLMs were configured with a temperature of 1.0. This setup more closely mimics each LLM’s “as-is” default behavior, improving the real-world relevance and comparability of results.

Dataset entries were initially written and executed in Polish, reflecting the linguistic and cultural context.

### 2.7. Statistical Analysis

Three separate sets of comparisons were performed using Pearson’s Chi-square tests with α = 0.05. Analysis 1 assessed the double-step prompt condition by comparing each LLM’s classifications to human expert judgments and by evaluating the dominant-category response against human annotations in pairs. Analysis 2 used the same pairwise comparison method for the Simplified prompt condition, testing all LLMs against the human reference and the dominant category. Analysis 3 conducted all possible pairwise comparisons between prompting strategies (Simple vs. Double-Step), all LLM outputs, human expert classifications, and the dominant response category, allowing a thorough evaluation of inter-rater agreement and prompt sensitivity.

Alongside Pearson’s Chi-square tests, we computed standard binary classification metrics (Accuracy, Precision, Recall, F1-score, and Specificity) to complement agreement-based reporting and better characterize asymmetric error patterns. All metrics were calculated against the expert-validated human reference across the whole dataset. “UNHEALTHY” was treated as the positive class, and “HEALTHY” as the negative class; metrics were derived from the confusion matrix (TP, FP, FN, TN). F1-score was calculated as $2\cdot \frac{Precision\cdot Recall}{Precision+Recall}$, and Specificity (true negative rate) as $Specificity=\frac{TN}{TN+FP}$.

**Figure 1:**
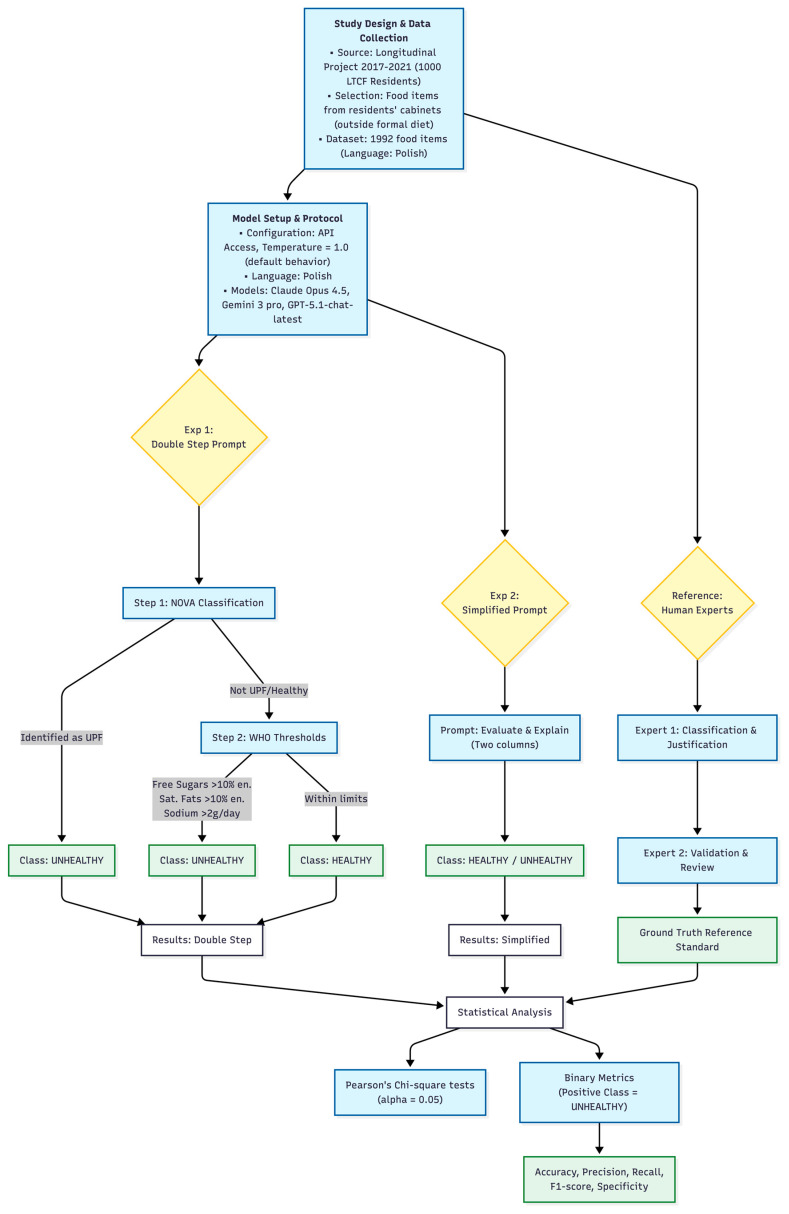
Study design and analytical workflow illustrating data selection (1992 food items), double-step NOVA+ World Health Organization (WHO) and simplified prompting of three large language models (LLMs), independent expert reference classification, and statistical evaluation using Pearson’s Chi-square tests and confusion-matrix-based metrics (Accuracy, Precision, Recall, F1-score, Specificity).

The complete study design is shown in the flowchart in Figure 1.

### 2.8. Use of Generative AI Tools

Generative AI tools were used in two ways in this study. First, three LLMs (Claude Opus 4.5, Gemini 3 Pro, and GPT-5.1-chat-latest) were accessed via API and used as classification engines, as described above. Second, an AI-assisted flowchart tool based on the Mermaid diagram syntax was used to draft the initial layout of the study flow diagram, which was then manually edited and finalized by the authors. All prompts, classification outputs, and graphical elements were reviewed and verified by the authors, who take full responsibility for the scientific content, visualizations, and interpretation.

## 3. Results

### 3.1. Overview of Classifications

Three advanced LLMs, Claude Opus 4.5, Gemini 3 pro, and GPT-5.1-chat-latest, were used to classify 1992 food items from LTCF residents’ personal food supplies. They employed both a structured double-step prompt (combining NOVA and WHO criteria) and a simplified single-step prompt. The classifications were compared to a validated human expert standard, established through independent review and correction by two experts. Of the 1992 items, human experts classified 41.9% (*n* = 835) as healthy and 58.1% (*n* = 1157) as unhealthy. The following sections detail inter-model agreement, model–human concordance, and the impact of prompting strategy on classification results.

### 3.2. Classification Agreement: Double-Step Prompt (NOVA + WHO Framework)

All three LLMs showed strong agreement with the human expert classification (90.3–91.3%), with Opus 4.5 achieving the highest level of agreement. The dominant category (consensus response across LLMs) showed comparable performance (91.1%), indicating robust inter-model consistency under structured prompting. Table 1 shows the classification agreement between the three LLMs and a human expert reference using the structured double-step prompt.

To complement the agreement breakdowns reported in Table 1 and Table 2 provides confusion-matrix-derived performance metrics (Accuracy, Precision, Recall, F1-score, and Specificity; UNHEALTHY as the positive class) against the expert-validated human reference. These metrics allow assessment of potential trade-offs between the detection of UNHEALTHY items (Recall) and the correct identification of HEALTHY items (Specificity) beyond total agreement.

Under the double-step NOVA+WHO framework, all three LLMs and the dominant consensus achieved high overall accuracy (0.904–0.913) with very high Recall for UNHEALTHY items (0.963–0.982), but at the cost of reduced Specificity (0.798–0.844), indicating a conservative tendency to classify borderline products as UNHEALTHY. The dominant classification performed similarly to the best individual models across all metrics, supporting the use of multi-model consensus as a robust, safety-oriented strategy.

### 3.3. Classification Agreement: Simplified Prompt

Under simplified prompting, GPT-5.1 achieved the highest individual LLM agreement with human classification at 93.6%, while the dominant category reached 94.2% concordance. Notably, all LLMs classified more items as healthy than the double-step framework, indicating reduced specificity in identifying ultra-processed or nutritionally inadequate foods. Table 3 summarizes classification results when LLMs were asked to evaluate items using simplified criteria without explicit NOVA or WHO thresholds.

To enable direct comparison with the double-step setting, Table 4 reports the same set of classification metrics for the simplified prompt, using the identical ground-truth distribution (*n* = 1992; HEALTHY *n* = 835; UNHEALTHY *n* = 1157).

Under the simplified prompt, all LLMs and the dominant consensus achieved higher overall accuracy (0.927–0.942) and markedly improved Specificity (0.897–0.951) compared with the double-step framework, while maintaining strong Recall for UNHEALTHY items (0.909–0.964). This pattern indicates that simplified prompting yields a more balanced trade-off between sensitivity and false-positive rates, with the dominant consensus again matching or slightly outperforming individual models across key metrics.

**Figure 2:**
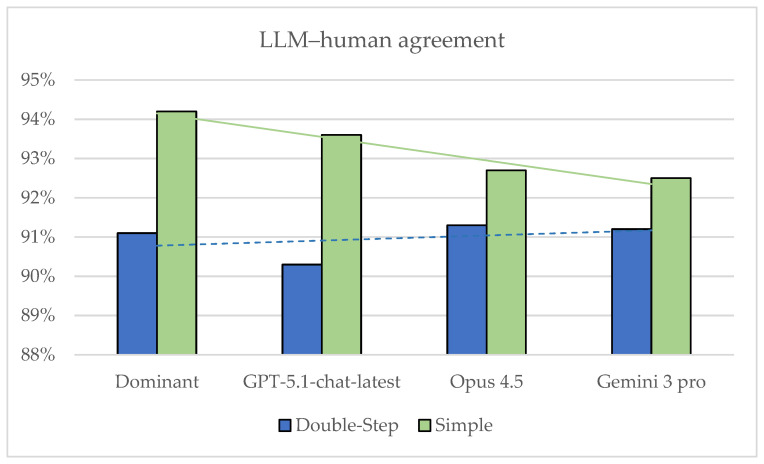
Overall agreement between three LLMs and the dominant consensus versus a human expert under double-step (NOVA+WHO) and simplified prompting.

To facilitate comparison across models and prompting strategies, Figure 2 summarizes total agreement with the human expert reference for each LLM and the dominant consensus under both conditions. 

### 3.4. Cross-Comparison of Prompt Strategies, LLMs, and Human Experts

Table 5 presents the results of the Pearson’s Chi-square test for all pairwise comparisons between double-step (NOVA + WHO) and simplified prompts, the three LLMs, the dominant categories, and human expert classification. All 36 comparisons were statistically significant (χ^2^ range: 1174.5–1897.1; *df* = 1; *p* < 0.001 for all), indicating apparent differences in classification patterns among evaluators and prompting methods.

The most significant divergence was found between WHO Gemini 3 pro and the dominant WHO category (χ^2^ = 1897.1), showing that Gemini 3 pro’s structured classification differed most from the consensus response. Conversely, the lowest chi-square value was observed between WHO GPT-5.1 and Simple Opus 4.5 (χ^2^ = 1174.5), indicating that these configurations, despite having different LLMs and prompts, produced more similar classification distributions.

Within-prompt comparisons showed significant differences between LLMs under both double-step (χ^2^ = 1608.3–1745.5) and simplified (χ^2^ = 1520.6–1641.3) conditions, indicating that LLMs evaluate nutritional criteria differently even when given the same prompt. Cross-prompt comparisons within each LLM revealed notable classification changes when switching from double-step to simplified prompting (χ^2^ = 1286.8–1478.5), confirming that prompt structure independently affects results regardless of LLM architecture. All LLMs under both prompting conditions differed significantly from human expert judgment (χ^2^ = 1296.6–1547.6), emphasizing ongoing gaps between AI-generated and expert-validated nutritional assessments.

## 4. Discussion

### 4.1. Inter-Model Agreement and Classification Consistency

This study critically evaluated three advanced LLMs, Claude Opus 4.5, Gemini 3 pro, and GPT-5.1, in automating the dietary classification of 1992 food items from residents in LTCFs using both a structured double-step prompt (incorporating NOVA and WHO frameworks) and a simplified single-step prompt. All three LLMs demonstrated high agreement with human expert classification under both prompting strategies (90.3–94.2%), yet Pearson’s Chi-square tests showed statistically significant differences in every pairwise comparison (χ^2^ = 1174.5–1897.1; *p* < 0.001), indicating notable variation in classification patterns across evaluators and prompts. These results show that AI-driven dietary assessment, even with the most advanced LLMs, reveals fundamental conflicts between processing-based and nutrient-threshold frameworks, with each LLM interpreting evidence-based criteria differently despite the same input [30,31,32,33].

### 4.2. Prompt Structure and Classification Specificity

In this study, the double-step prompt was clearly grounded in two evidence-based frameworks: NOVA for processing level and WHO nutrient thresholds for free sugars, saturated fat, and sodium, thereby ensuring a highly rigorous, rule-based classification process. Although NOVA has known limitations, including inconsistent product grouping and partial misalignment with nutrient-based risk at the individual product level, it remains a commonly used tool in epidemiological and public health research. It provides a transparent way to define ultra-processing [21,22]. With such a strict, framework-driven prompt, it was reasonable to expect that agreement between LLMs would be higher and more consistent, since each LLM was limited to following the same clearly defined decision rules rather than relying on internal heuristics.

Prompt design remained a key factor in classification results, with cross-prompt comparisons showing significant changes within each LLM when switching from double-step to simplified prompting (χ^2^ = 1286.8–1478.5; *p* < 0.001). Under the structured double-step approach, LLMs labeled 64.4% of items as unhealthy, reflecting the strict use of the NOVA ultra-processing criteria and the WHO nutrient thresholds. In contrast, the simplified prompt resulted in only 56.6% of items being classified as unhealthy, indicating lower specificity. Interestingly, all LLMs under simplified prompting had higher agreement with human experts (92.5–93.6% vs. 90.3–91.3%), but this increase in agreement came at the expense of lower alignment with evidence-based nutritional frameworks. These findings confirm that binary, framework-based prompts improve reproducibility and adherence to established dietary guidelines, whereas simplified prompts may better mimic overall human judgment but compromise methodological rigor [34,35].

These patterns are reflected in the confusion-matrix-based metrics reported in Table 2 and Table 4, where the double-step NOVA+WHO prompt yields very high Recall but lower Specificity for UNHEALTHY items. In contrast, the simplified prompt provides higher overall Accuracy and a more balanced recall–specificity profile across all LLMs. This supports the interpretation that simplified prompting tends to align more closely with general human judgment, while the double-step framework maintains stricter adherence to guideline-based risk thresholds.

### 4.3. Linguistic Considerations: Polish Language and AI Capabilities

A key methodological strength of this study is the use of Polish-language food product descriptions directly from the LTCF database, which maintains the authentic linguistic and cultural context of the original data. Choosing to keep the data in its native language is essential because Polish, a morphologically rich Slavic language with clear case marking, flexible word order, and complex derivational morphology, has been demonstrated in recent multilingual benchmarking efforts to rank among the top-performing languages for LLM instruction-following and complex reasoning tasks, often outperforming English [26]. The morphosyntactic specificity of Polish product names and ingredient descriptions seems to improve LLM parsing and semantic disambiguation, especially in nutrition contexts that need precise separation of processing methods and ingredient types.

Notably, translating the Polish dataset into English or other languages could have changed classification results, as linguistic nuances embedded in product terminology, such as culturally specific food categories, preparation methods, and colloquial ingredient descriptions, may experience semantic loss or reinterpretation during translation [36]. These findings highlight that language is likely not just a neutral channel for data transfer but an active factor influencing AI model performance in dietary assessment, with implications for developing multilingual nutrition tools and the need for linguistically validated, culture-specific benchmark datasets [37,38,39].

### 4.4. Conservative Classification Bias and Risk Mitigation Strategies

All evaluated LLMs showed a cautious safety bias, especially with structured prompts, often defaulting to “unhealthy” classifications when product ingredient details were incomplete or unclear. Although this careful approach might be overly cautious at times, it is beneficial from a patient-risk perspective because false negatives (incorrectly labeling unhealthy items as healthy) can cause more harm than false positives in clinical and public health settings.

The elevated Recall and reduced Specificity observed for the double-step prompt in Table 2 are consistent with this conservative bias, whereas the simplified prompt results in a more balanced error pattern, as shown in Table 4. The main category across LLMs (consensus classification) achieved 91.1–94.2% agreement with human experts, suggesting that multi-model voting strategies can help reduce individual LLM biases while maintaining a conservative risk posture. However, this cautiousness also highlights the limits of automated analysis when input data lacks detail, emphasizing the need for structured data collection and expert review for ambiguous cases [4,5,40].

### 4.5. Practical Efficiency: Workflow Assisted by LLM Versus Manual Classification from Scratch

From a pragmatic perspective, the high baseline accuracy of LLMs (90.3–94.2% agreement with human experts) directly yields significant time and resource savings in real-world dietary assessment workflows. Instead of requiring human experts to classify 1992 items from scratch, a process that demands extensive nutritional knowledge, familiarity with the framework, and sustained mental effort, practitioners can review and correct a pre-classified dataset in which 90–94% of entries are already correct. This “review-and-refine” approach significantly lessens the workload for experts, allowing them to focus on the 6–10% of ambiguous or borderline cases where nuanced judgment is most needed [41].

For large-scale epidemiological studies, clinical trials, or population surveillance efforts involving tens of thousands of food entries, this efficiency improvement enables the practical implementation of rigorous nutritional classification protocols that would otherwise be extremely labor-intensive [42]. Importantly, even with a conservative LLM bias requiring expert correction of over-classified “unhealthy” items, the cognitive load of validation remains much lower than that of initial classification, as experts only need to assess the LLM’s reasoning rather than research each product from scratch. This workflow efficiency highlights the practical value of LLM-assisted dietary assessment as a scalable support tool rather than a substitute for human expertise [3].

### 4.6. Human Expertise and the Importance of Expert Oversight

Our findings strongly emphasize the essential role of human expert validation alongside LLM-generated classifications. Despite high overall agreement (90.3–94.2%), all LLMs under both prompting conditions differed significantly from human expert judgment (χ^2^ = 1296.6–1547.6; *p* < 0.001), with disagreements mainly occurring in borderline cases involving minimally processed foods with moderate WHO threshold exceedances or ambiguous NOVA classifications. The two-expert validation process used in this study, where a second expert reviewed and corrected initial classifications, proved crucial for establishing a highly accurate reference standard. While LLMs can offer valuable guidance to lay users in daily dietary choices, especially given their conservative bias, large-scale applications such as epidemiological surveillance, clinical trials, and population-level dietary assessments require mandatory expert review to validate AI outputs and incorporate detailed contextual judgment [43,44]. This layered approach balances the scalability and accessibility of AI-assisted assessment with the interpretive power and safety provided by human expertise.

### 4.7. Limitations and Future Directions

Several limitations should be considered. First, the accuracy of LLMs depends on the completeness of product descriptions and the reliability of the training data, with classification errors more likely to impact ambiguous or poorly described items [4]. Second, language-specific benefits seen in Polish may not fully apply to simpler or less inflected languages, requiring thorough multilingual testing [45]. Third, current LLMs lack actual multimodal reasoning, preventing direct analysis of product packaging, nutrition labels, or ingredient photos, which would significantly improve real-world usefulness [46].

A further limitation is that, although the data came from residents of LTCFs, human experts were explicitly instructed to classify products “holistically and generally” rather than tailoring their judgments to LTCF-specific dietary needs. Consequently, some items considered acceptable for a generally healthy adult population might not fully reflect the nutritional needs, comorbidities, or texture-modified diets typical of LTCF residents, which should be considered when assessing the real-world relevance of these findings.

Future research should focus on developing multimodal AI frameworks that integrate text, images, and structured nutrient data; creating standardized benchmark datasets with expert-validated ground truth across diverse populations; and establishing robust hybrid workflows that combine AI efficiency with mandatory expert review thresholds triggered by classification uncertainty scores [47]. Importantly, future studies must also address linguistic bias by conducting parallel evaluations across multiple source languages, maintaining original product terminology rather than relying on translated datasets, and measuring language-specific performance differences to promote equitable deployment of AI tools across culturally and linguistically diverse groups. Additionally, longitudinal validation studies assessing LLM performance across product reformulations and changing dietary guidelines will be essential to ensure ongoing accuracy and clinical relevance [48,49].

## 5. Conclusions

This study shows the significant potential of LLMs for scalable, efficient dietary assessment in real-world clinical and public health settings. By systematically evaluating three advanced LLMs against expert-validated classifications of 1992 food items using both structured (NOVA + WHO) and simplified prompting strategies, we found high overall agreement (90.3–94.2%), confirming that current LLMs achieve high agreement with human experts in automated nutritional classification. The practical efficiency of LLM-assisted workflows allows experts to review and correct pre-classified datasets rather than start from scratch, significantly reducing labor while maintaining high-quality standards. This makes large-scale dietary assessment projects more feasible and cost-effective. Additionally, languages with rich linguistic features, like Polish, provide measurable advantages in LLM reasoning and classification accuracy, with multi-model consensus strategies partially reducing individual biases while maintaining conservative risk assessment [50].

Despite notable progress, the significant differences observed between LLMs and humans (χ^2^ = 1174.5–1897.1; *p* < 0.001 for all comparisons) confirm that AI-based nutritional assessment remains influenced by prompt design, LLM architecture, and language context. Current LLMs still struggle with incomplete product data, integrating multimodal information (such as nutrition labels and ingredient photos), and fully mimicking nuanced clinical judgment, especially for borderline cases involving minimally processed foods or moderate WHO threshold exceedances [51,52,53]. Translation-related semantic loss poses a significant bias risk in multilingual settings, and performance varies across languages, which requires careful validation before cross-cultural use. Human expert validation remains essential for ambiguous cases and high-stakes situations, as LLMs cannot yet resolve classification uncertainties independently [36].

As digital health progresses, four key strategic priorities will shape the most effective dietary assessment workflows. First, develop multimodal AI frameworks that integrate text, images, and structured nutrient data to improve real-world use and reduce reliance on manual product descriptions. Second, create standardized multilingual benchmark datasets that maintain source-language authenticity to measure language-specific performance differences and promote fair AI deployment across diverse cultures and languages. Third, implement robust hybrid workflows that merge AI scalability with mandatory expert review thresholds triggered by classification uncertainty scores, balancing efficiency and quality. Fourth, conduct longitudinal validation studies to evaluate LLM performance over product reformulations and changing dietary guidelines, ensuring ongoing accuracy and clinical relevance [54,55].

The demonstrated efficiency improvements and high baseline accuracy (90.3–94.2%) of current LLMs support the use of LLM-assisted dietary classification as a valuable tool for nutrition research and public health monitoring while ensuring scientific rigor through required human oversight validation. Structured, evidence-based prompts improve reproducibility and adherence to the framework but may reduce flexibility in holistic judgment, requiring context-dependent prompt selection aligned with study goals.

When interpreting these findings, it is also important to recognize that the expert reference was defined for a generally healthy adult diet, while the underlying data came from LTCF residents, meaning that some classifications may not fully capture population-specific nutritional needs or clinical aspects constraints. A balanced approach that combines AI scalability with expert oversight, transparent uncertainty measurement, and language-aware design principles will be key to providing personalized, reliable nutritional guidance that can adapt to complex real-world scenarios and diverse populations.

## Figures and Tables

**Table 1:** Pairwise agreement between large language models LLMs and human experts using a double-step prompt (NOVA + WHO criteria). Values represent the percentage of the total dataset (N = 1992).

Comparison	Healthy–Healthy	Healthy– Unhealthy	Unhealthy–Healthy	Unhealthy– Unhealthy	Total Agreement
GPT-5.1 vs. Human	33.4%	1.2%	0.8%	56.9%	**90.3%**
Opus 4.5 vs. Human	35.4%	2.2%	0.9%	55.9%	**91.3%**
Gemini 3 pro vs. Human	34.2%	1.1%	1.1%	57.0%	**91.2%**
Dominant vs. Human	34.3%	1.3%	0.4%	56.8%	**91.1%**

**Table 2:** Confusion-matrix-derived performance metrics for the structured double-step (NOVA + WHO) prompt against the expert-validated human reference (N = 1992; HEALTHY *n* = 835; UNHEALTHY *n* = 1157). UNHEALTHY was treated as the positive class.

LLM	TP	FP	FN	TN	Accuracy	Precision	Recall	F1-Score	Specificity
GPT-5.1-chat-latest	1134	169	23	666	0.904	0.870	0.980	0.922	0.798
Claude Opus 4.5	1114	130	43	705	0.913	0.895	0.963	0.928	0.844
Gemini 3 pro	1136	153	21	682	0.913	0.881	0.982	0.929	0.817
Dominant vs. Human	1131	152	26	683	0.910	0.881	0.977	0.927	0.818

**Table 3:** Pairwise agreement between LLMs and human experts using a simplified single-step prompt. Values represent the percentage of the total dataset (N = 1992).

Comparison	Healthy–Healthy	Healthy– Unhealthy	Unhealthy–Healthy	Unhealthy– Unhealthy	Total Agreement
GPT-5.1 vs. Human	37.6%	2.1%	3.8%	56.0%	93.6%
Opus 4.5 vs. Human	39.9%	5.3%	3.2%	52.8%	92.7%
Gemini 3 pro vs. Human	39.6%	4.9%	2.6%	52.9%	92.5%
Dominant vs. Human	39.8%	3.7%	2.2%	54.4%	94.2%

**Table 4:** Confusion-matrix-derived performance metrics for the simplified single-step prompt against the expert-validated human reference (N = 1992; HEALTHY *n* = 835; UNHEALTHY *n* = 1157). UNHEALTHY was treated as the positive class.

LLM	TP	FP	FN	TN	Accuracy	Precision	Recall	F1-Score	Specificity
GPT-5.1-chat-latest	1115	86	42	749	0.936	0.928	0.964	0.946	0.897
Claude Opus 4.5	1052	41	105	794	0.927	0.962	0.909	0.935	0.951
Gemini 3 pro	1060	46	97	789	0.928	0.958	0.916	0.937	0.944
Dominant	1084	43	73	792	0.942	0.962	0.937	0.949	0.948

**Table 5:** Pearson’s Chi-square test results for all pairwise comparisons (N = 1992; *df* = 1; *p* < 0.001 for all).

	WHO GPT-5.1	WHO Opus 4.5	WHO Gemini 3 Pro	WHO Dominant	Simple GPT-5.1	Simple Opus 4.5	Simple Gemini 3 Pro	Simple Dominant	Human
WHO GPT-5.1	—	1608.3	1745.5	1838.0	1478.5	1174.5	1207.2	1282.4	1296.6
WHO Opus 4.5		—	1661.7	1749.0	1525.0	1331.3	1287.0	1347.1	1347.4
WHO Gemini 3 pro			—	1897.1	1587.8	1286.8	1349.2	1401.3	1354.5
WHO Dominant				—	1548.6	1250.4	1305.2	1363.6	1338.8
Simple GPT-5.1					—	1551.3	1520.6	1694.1	1500.7
Simple Opus 4.5						—	1641.3	1827.6	1449.0
Simple Gemini 3 pro							—	1798.9	1456.1
Simple Dominant								—	1547.6
Human									—

Note 1: WHO = double-step prompt (NOVA + WHO criteria); Simple = simplified single-step prompt; Dominant = consensus category across LLMs. All χ^2^ values are significant at *p* < 0.001.

## Data Availability

The data that support the findings of this study are available from the corresponding author upon reasonable request due to legal reasons.
